# Management of methylmalonic acidemia (MMA) with *N*‐carbamylglutamate: A case report from Italy

**DOI:** 10.1002/mgg3.2073

**Published:** 2022-11-04

**Authors:** Flavia Tubili, Francesca Pochiero, Maria Rosaria Curcio, Elena Procopio

**Affiliations:** ^1^ Metabolic and Neuromuscular Unit, Meyer Children Hospital University of Florence Florence Italy

**Keywords:** Carglumic acid, Italy, methylmalonic acidemia, *N*‐carbamylglutamate

## Abstract

**Background:**

Methylmalonic acidemia (MMA) is an inborn error of metabolism whose optimal management, especially in the long‐term remains to be established.

**Methods:**

We describe the case of a child with MMA *mut* ^0^ who was in a cycle of episodes of decompensation and hospitalization when we started to use carglumic acid (CA), a well‐known adjunctive therapy to standard care for the treatment of acute hyperammonemia due to MMA.

**Results:**

Using the lowest effective therapeutic dose of CA and adjusting the patient's diet with caloric and protein intake adequate for her age and pathology, we managed to keep ammonium levels within the normal range, and to ensure a normal growth pattern.

**Conclusion:**

The present case adds further confirmation of the long‐term management of MMA using CA, focusing on the long duration of follow up and on the use of a lower dose of CA in real life settings.

## Graphical abstract

We present the case report of a patient with frequently decompensated methylmalonic acidemia (MMA) who was successfully treated with carglumic acid (Carbaglu®, CA). The efficacy and safety of long‐term CA in the management of MMA have been confirmed in a prospective, randomized controlled trial and our report provides additional real‐world evidence on the ability of CA to improve the quality of life of patients with this rare disease.

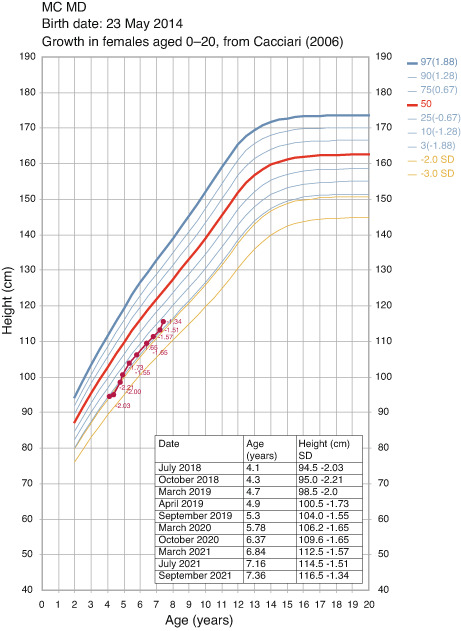

## 1 INTRODUCTION

Methylmalonic acidemia (MMA OMIM #251000) is an autosomal recessive inborn error of metabolism due to a complete (*mut* ^0^) or partial (*mut* ^−^) deficiency of the mitochondrial enzyme methylmalonyl‐CoA mutase (*MMUT gene*) or by deficient synthesis of the MUT‐cofactor adenosylcobalamin. It is a rare disorder that can cause significant morbidity and mortality and its incidence varies considerably worldwide (Haijes et al., 2019). A recent meta‐analysis reported rates among newborns ranging from 0.79 per 100,000 in Asia‐Pacific countries to 6.04 per 100,000 in the Middle East and North Africa (MENA) (Almási et al., 2019). In Europe, the increasing use of expanded newborn screening programs has permitted presymptomatic detection, with a study from Spain in 71,595 newborns identifying 10 cases of MMA (13.97 per 100,000) (Castiñeras et al., 2019; Juan‐Fita et al., 2012). Similarly, in Italy, three cases of MMA were detected among 45,466 neonates (6.60 per 100,000) (Scolamiero et al., 2015).

In the neonatal onset form of MMA, symptoms start as early as the second day of life with acute deterioration of clinical condition, metabolic acidosis, and hyperammonemia, progressing to coma and death if untreated (Baumgartner et al., 2014). Acute intermittent late‐onset MMA may present at any age (infancy, childhood, or later) and it has a more heterogeneous clinical picture. The clinical course of MMA is characterized by periods of stability and intermittent metabolic decompensation, usually associated with intercurrent infections and stress. The accumulation of toxic compounds (organic acids, ammonia) can cause severe clinical symptoms including prolonged vomiting and diarrhea, dehydration, hypotonia, irritability, and lethargy. Long‐term complications include failure to thrive, intellectual disability, progressive renal disease, pancreatitis, and cardiac dysfunction.

The lack of a universal consensus on how to diagnose and treat patients with MMA resulted in the development of guidelines by international experts and the first of these was published in 2014 (Baumgartner et al., 2014) with an update published 6 years later (Forny et al., 2021).

As the long‐term survival in MMA has been significantly improving over the years, effective management of long‐term complications is now even more important.

We present the case report of a patient with frequently decompensated MMA who required multiple hospitalizations during the first 6 years of her life.

## 2 CASE PRESENTATION

A female Caucasian infant born at term after an uncomplicated pregnancy to referred non‐consanguineous parents. An elective cesarean section was performed due to nonengagement at 39 + 5 weeks. Birth weight was 2679 g (standard deviation score − 1.42; 8th percentile, small for gestational age, SGA) and her Apgar scores at 1 and 5 min after birth were 7 and 9, respectively. Twenty‐four hours later she became lethargic, and her feeding decreased, resulting in dehydration and progressive weight loss with generalized hypotonia and jaundice. She was admitted to the intensive care unit in poor general condition and on the 5th day of life weighed 2160 g (19% loss from birth). Blood tests showed the presence of metabolic acidosis and hyperammonemia (294 μmoL/L). The elevated levels of propionylcarnitine (C3 32.5 μmoL/L, normal range 0.04–3.18), of free plasmatic methylmalonic acid and of urinary methylmalonic acid (5477 μmol/mol of creatinine, normal value up to 6), with normal levels of homocysteine, allowed the diagnosis of MMA. Molecular analysis of the *MMUT* gene showed the pathogenic homozygous variant NM_000255.4:c.628C > T p.(Arg228*) in exon 3, associated with MMA *mut* ^0^. The patient's clinical file from the previous hospital noted that she was treated with arginine, carnitine, sodium bicarbonate, and glucose infusions. Enteral feeding with a low protein and precursor‐free amino acid mixture started when she was 10 days old showed a clinical improvement and growth improved. The patient was discharged after nearly 50 days hospitalization with a low‐protein diet and carnitine therapy (100 mg/kg/day). A second hospitalization took place aged 2 months (6 days after the previous discharge) for acute decompensation with metabolic acidosis during gastroenteritis. It was followed by a third hospitalization 4 months later (aged 6 months) for hypoalimentation, gastroenteritis, hyperglycemia, metabolic acidosis, and sepsis. She remained relatively well until she was hospitalized aged 4 years and 1 month for severe metabolic acidosis during intercurrent illness. Her clinical records show that oral l‐arginine (80 mg/kg/day) was introduced.

She was evaluated for the first time in our center aged 4 years and 2 months. She had a very difficult weaning that started at 18 months of age, with refusal of food. At the time of the visit, her diet was unvaried and consisted mainly of milk and biscuits: caloric intake 81 kcal/kg/day and controlled protein intake 1.2 g/kg/day (15.5 g/day), 12.88 g/day from natural proteins and 2.62 g/day of synthetic proteins, in four meals distributed approximately every 6 h. Her height‐weight gain was within the lower limits: 12.8 kg (<3rd percentile), height 94.5 cm (3rd percentile), HC (head circumference) 49.5 cm (50th percentile). She had normal psychomotor development.

As her plasma arginine values were in the low‐normal range and given published evidence on the role of arginine in supporting normal growth, we decided to continue with therapy started at her previous clinic (Molema et al., 2019).

**FIGURE 1:**
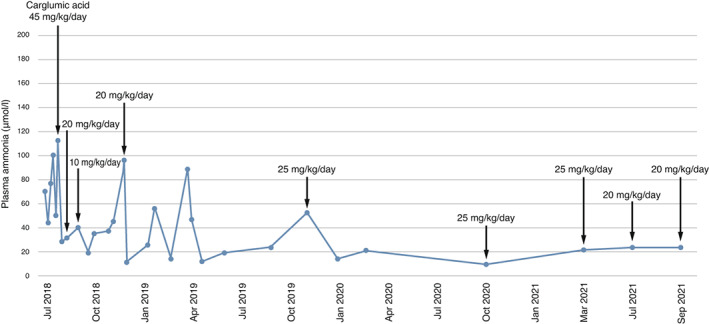
Plasma ammonia levels from July 2018 to September 2021 before and after treatment with carglumic acid (Carbaglu®, CA)

**FIGURE 2:**
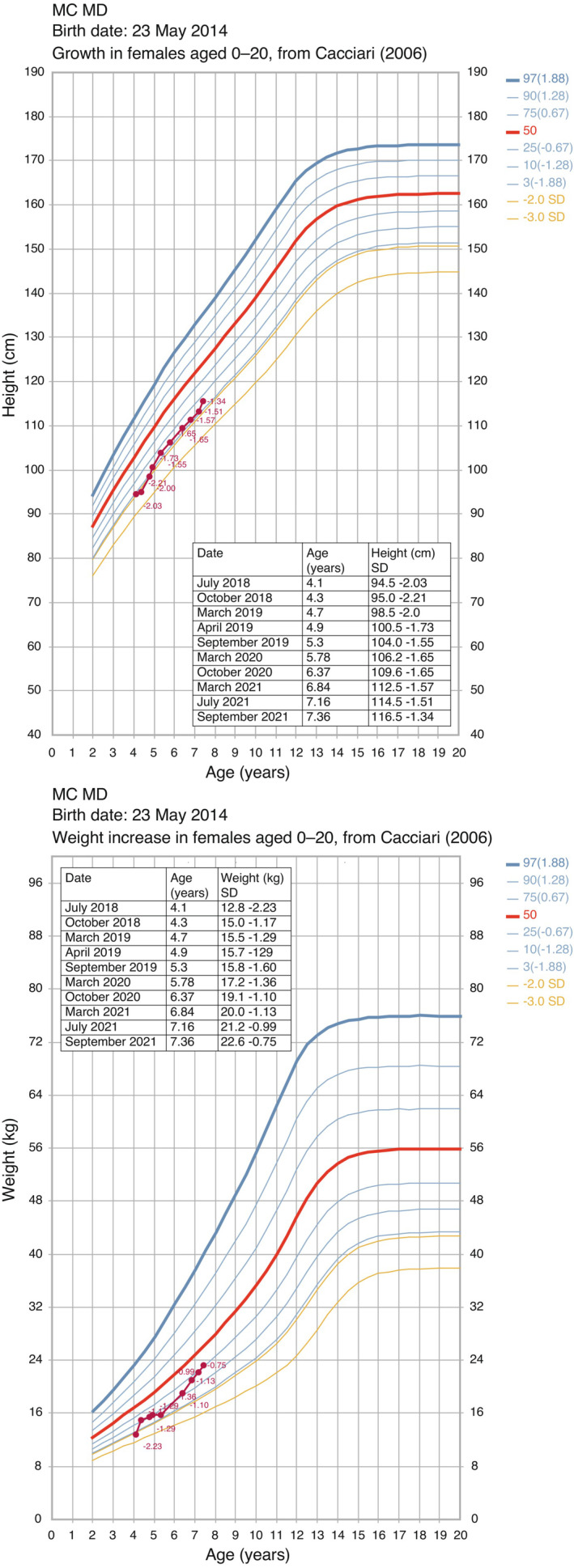
Growth charts for height and weight (a and b) of patient from July 2018 to September 2021. Curves for 3rd, 25th, 50th, 75th and 97th are reported in each graph (growth charts reproduced from: Cacciari E, et al. Italian cross‐sectional growth charts (2 to 20 yr). J Endocrinol invest. 2006; 29(7): 581–593)

Her therapy was reviewed, and a series of changes were made to break the cycle of episodes of decompensation and hospitalization. Carglumic acid (Carbaglu®, CA) was introduced (45 mg/kg/day) to treat persistent mild hyperammonemia (69 μmoL/L, normal value <50), (Figure 1). This gradually allowed us to modify her diet, increasing the number of meals (from 4 to 5), introducing solid food and maintaining an appropriate caloric (82 kcal/kg/day) and protein intake for her age (0.87 g/kg/day). Moreover, we could discontinue the precursor‐free amino acid mixture. Her feeding improved and her weight increased to 14.4 kg (3rd percentile). Due to mild hypokalemia, therapy with potassium aspartate was introduced. She remained stable and 3 weeks later the dosage of CA was reduced to 20 mg/kg/day and to 10 mg/kg/day a week later. After 5 months, she was hospitalized for persistent vomiting and poor feeding, necessitating a glucose infusion. The dosage of CA was increased back to 20 mg/kg/day as there was a slight increase in ammonia levels (86 μmoL/L). Allopurinol therapy was introduced for hyperuricemia (7.6 mg/dL, normal range 1.4–4.1). As per the pathology, she developed moderate renal failure (creatinine 1.13 mg/dL normal range 0.4–0.7; glomerular filtration rate 43 ml/min). Her condition remained relatively stable, she gained weight and height, but she showed persistent metabolic acidosis necessitating an increase in sodium bicarbonate dosage (0.7 mEq/kg in four administrations/day). At the routine visits (aged 6 years 5 months; 6 years 8 months; 7 years 1 month and 7 years and 4 months), she was adhering well to therapy and her condition continued to improve (Figure 2a,b). The Weschsler Preschool and Primary Scale of Intelligence Third edition (WPPSI‐III) performed at 6 years and 3 months showed she had an intelligence quotient (IQ) of 83 (13th percentile, CI 95%:76–90), a verbal IQ of 104 and a performance IQ of 70. She follows a diet with safe levels of calories and protein (0.87 g/kg/day), has a good appetite, manages to finish meals, and adheres well to her therapy regimen (CA 20 mg/kg/day, bioarginine, carnitine, allopurinol, sodium bicarbonate, potassium aspartate, and multivitamins).

## 3 DISCUSSION

Updated guidelines on the management of MMA represented a major step forward for patients with MMA (Forny et al., 2021). Early diagnosis and timely treatment of MMA are essential to improve survival and reduce morbidity in MMA patients. We know that it is critical during acute decompensation episodes to rapidly normalize blood ammonia levels to avoid neurological damage and associated complications, but it is likewise mandatory to achieve metabolic stability defined as the absence of hospitalization and exacerbation of disease signs and symptoms. CA, a carbamoyl phosphate synthetase 1 (CPS 1) activator and a structural analogue of *N*‐acetylglutamate is indicated in pediatric and adult patients as adjunctive therapy to standard of care for the treatment of acute hyperammonemia due to MMA or propionic acidemia (PA). Studies have shown that it effectively and rapidly reduces hyperammonemia when added to standard treatment for acute metabolic decompensation both in newborns and older patients with MMA (Chakrapani et al., 2018; Valayannopoulos et al., 2016). Although the role of acute treatment with CA has been demonstrated further systematic studies are required to prove efficacy with long‐term treatment. In fact, the updated guidelines state whether CA can consistently reduce the number of metabolic decompensations needs to be further studied. It has been tried to anapleurotically support Krebs cycle function by providing citrate, which was associated with reduced number of hospitalizations in three PA patients (Forny et al., 2021). In addition, data on its efficacy and safety as a long‐term therapy are lacking.

A case report published in 2018 discussed a patient with PA who had 78 hospital admissions for hyperammonemia over a 9‐year period but when treatment with CA was started (100 mg/kg/day for 6 months, reduced to 50 mg/kg/day for a total duration of treatment of 6 years), required only two admissions for decompensation in the first year of therapy and none subsequently (Tummolo et al., 2018).

Burlina et al. conducted a retrospective, observational review of patient medical records to assess the clinical experience of eight patients with PA (*n* = 4) and MMA (*n* = 4) who were experiencing frequent progressive episodes of metabolic decompensation and who had pathological levels of ammonia (Burlina et al., 2016). In the year before starting continuous treatment with CA (50 mg/kg/day) all patients had at least three decompensation episodes and one patient with PA had 11 episodes. All MMA patients and one with PA required hospitalization for a metabolic decompensation during this period. Following treatment with CA (50 mg/kg/day) for 7–16 months, the number and severity of metabolic decompensation episodes decreased. Patients had improved appetite, gained weight, and increased their natural protein intake. The authors concluded that in addition to short‐ and long‐term benefits in the acute treatment of hyperammonemia, CA appears to be an effective and well tolerated long‐term treatment in patients with severe PA and MMA (Burlina et al., 2016).

Another more recent single‐center, retrospective trial investigated 21 patients (11 with MMA and 10 with PA) where CA was used as an ammonia scavenger for an average of 23 months (3–51 months) (Kiykim et al., 2021). It was started during the hospital stay due to metabolic decompensation in 11 patients and at the outpatient clinic in 10 patients due to persistent mild hyperammonemia. The average CA dosage was 85 mg/kg/day (range 12.5–250 mg/kg/day). There was a significant decrease in plasma ammonia levels with long term CA treatment in comparison with pre‐CA treatment period (*p* = .021). Hyperammonemia episodes requiring hospitalization decreased with CA treatment and hyperammonemia episodes were successfully treated with CA. CA was an effective and safe treatment for the chronic management of hyperammonemia in patients with PA and MMA. Recently, a newborn exhibiting early onset PA with severe hyperammonemia was effectively managed in the long‐term with CA treatment: she did not decompensate and hospitalization was not required in the 26 months of follow‐up (Kido et al., 2020).

In a prospective, multicenter, randomized, controlled clinical trial, patients aged ≤15 years with confirmed MMA were followed‐up for 2 years (Nashabat et al., 2019). The objective of the study was to compare the efficacy of adding CA (50 mg/kg/day in divided doses, twice daily) to standard treatment in patients with PA and MMA. Results, of what the authors describe as the first prospective clinical trial to evaluate the efficacy of long term of CA, confirmed that CA is well tolerated and significantly reduces the number of hospital admissions due to hyperammonemia in patients with PA and MMA (Alfadhel et al., 2021). Thirty‐eight patients (21 received CA and 17 standard therapy) were included in the study and on the primary efficacy endpoint, a mean of 6.31 emergency room admissions was observed for the CA arm, compared with 12.76 for standard treatment, with a significant difference between the groups (*p* = .0095).

There is now a substantial body of evidence showing that CA combined with standard treatments, prevents metabolic decompensation and hence hospitalization as well as improving quality of life.

The overall management of our patient presented several challenges and limitations. She had been hospitalized many times and our objective was to break the cycle of episodes of decompensation and hospitalization. Treatment with CA and dietary adjustments to maintain a caloric and protein intake as per her age/pathology ensured ammonium levels were kept within the normal range, allowing a regular weight/height increase.

The long duration of follow up and use of a lower dose of CA (minimum effective dose) than that discussed in guidelines make our results of particular interest to healthcare professionals in their everyday real world clinical practice. In the long term, it may not be necessary to increase the dose according to body weight, as our case demonstrated adequate metabolic control can be achieved with lower daily doses while maintaining plasma ammonia levels within the normal range and clinical condition (nutritional requirements, protein intake, growth parameters). As well as additional evidence of the effectiveness of CA therapy in both acute and long‐term management of MMA.

## AUTHOR CONTRIBUTIONS

All authors contributed to the research, development, and content of the manuscript and all authors attest that they meet the current ICMJE criteria for Authorship.

## FUNDING INFORMATION

Supported by an unrestricted grant from Recordati Rare Diseases.

## CONFLICT OF INTEREST

The authors report no conflicts of interest.

## PATIENT CONSENT

The parents of the patient were informed that data regarding the case would be presented for publication, and they consented.

## Data Availability

Data sharing not applicable to this article as no datasets were generated or analysed during the current study.
